# Identification of *Fusarium oxysporum* Causing Leaf Blight on *Dendrobium chrysotoxum* in Yunnan Province, China

**DOI:** 10.3390/life14030285

**Published:** 2024-02-20

**Authors:** Jun Yang, Waqar Ahmed, Jinhao Zhang, Shunyu Gao, Zhenji Wang, Haiyan Yang, Xuehui Bai, Kai Luo, Chengdong Xu, Guanghai Ji

**Affiliations:** 1 College of Resources, Environment and Chemistry, Chuxiong Normal University, Chuxiong 675000, China; yangjun@cxtc.edu.cn (J.Y.); gsy88@cxtc.edu.cn (S.G.); wangzj@cxtc.edu.cn (Z.W.); haiyanyang@cxtc.edu.cn (H.Y.); 2 State Key Laboratory for Conservation and Utilization of Bio-Resources, Yunnan Agricultural University, Kunming 650201, China; ahmed.waqar1083@yahoo.com (W.A.); jinhaoynau@163.com (J.Z.); 3 Dehong Insitute of Tropical Agriculture in Yunnan Province, Ruili 678600, China; baixue0059@163.com (X.B.); luokaiyss@163.com (K.L.)

**Keywords:** Koch’s postulates, pathogenicity, morphological characteristics, phylogenetic analysis, internal transcribed spacer (ITS)

## Abstract

Leaf-blight disease caused by the *Fusarium oxysporum* is an emerging problem in *Dendrobium chrysotoxum* production in China. Symptoms of leaf blight were observed on seedlings of *D. chrysotoxum* cultivated in a nursery in Ruili City, Yunnan Province, China. In this study, we isolated the *Fusarium* sp. associated with leaf-blight disease of *D. chrysotoxum* from the diseased seedlings. A pathogenicity test was performed to fulfill Koch’s postulates to confirm the pathogenicity of isolated strains and identified using morphological and molecular techniques. The results revealed that all four isolated *Fusarium* sp. isolates (DHRL-01~04) produced typical blight symptoms followed by marginal necrosis of leaves on the *D. chrysotoxum* plants. On the PDA medium, the fungal colony appeared as a white to purplish color with cottony mycelium growth. Microconidia are oval-shaped, whereas macroconidia are sickle-shaped, tapering at both ends with 2–4 septations. The phylogenetic trees were construed based on internal transcribed spacer (*ITS*), translation elongation factor (*EF-1α*), and RNA polymerase subunit genes *RPB1* and *RPB2* genes, respectively, and blasted against the NCBI database for species confirmation. Based on the NCBI database’s blast results, the isolates showed that more than 99% identify with *Fusarium oxysporum*. To our knowledge, this is the first comprehensive report on the identification of *Fusarium oxysporum* as the causal agent of *Dendrobium chrysotoxum* leaf blight in Yunnan Province, China, based on morphological and molecular characteristics.

## 1. Introduction

Orchidaceae is the chief family of plants, with more than 25,000 plant species worldwide [1]. They are also some of the most vulnerable flowering plants, as many genera are endangered, and almost all are at risk of habitat damage and overclassification [2]. The *Dendrobium* represents one of the most important genera of the *Orchidaceae*, first identified by Olaf Swartz in 1799 AD, which comprises approximately 1500 to 2000 species [3]; among them, 74 species and two varieties are native to China [4]. Genus *Dendrobium* contains species with high medicinal and ornamental values, such as the *D. chrysotoxum* Lindl. [5], *D. fimbriatum* Hook [6], *D. nobile* Lindl. [7], and *D. officinale* Kimura et Migo [8]. In China, *Dendrobium* species have traditionally been used as first-rate medicinal herbs to treat various disorders, such as stomach nourishment and diabetes [9]. The stems and leaves of *Dendrobium* are rich sources of polysaccharides, and one class that contains mannans is used in drugs and has a high market value [10]. *Dendrobium* plant parts, especially leaves and stems, are used in traditional Chinese medicine (TCM) to control diabetes, rheumatoid arthritis, obesity, and many other diseases and are enlisted in the famous 18 ancient TCM [11]. In China, the history of *Dendrobium* was written about 2300 years ago as “Shen Nong’s Herbal Classic (the Eastern Han Dynasty)” TCM, and it is cultivated as both a medicinal and ornamental plant [12]. Among the *Dendrobium* species, *D. officinale* has the foremost therapeutic properties and is used as “Shihu” in TCM. Many species of *Dendrobium* are the sources of tonic for astringents, analgesics, antipyretics, antioxidants, antimicrobials, antidiabetics, anticancers, antiinflammatories, antimetastases, and antiangiogenetics because they have alkaloids, aromatic compounds, sesquiterpenoids, and polysaccharides as main components [13].

*D. chrysotoxum* is known as a “fried egg” orchid and is widely cultivated as an ornamental and medicinal plant [14]. *D. chrysotoxum* has high medicinal value due to its enrichment of various types of chemical constituents, such as bibenzyls, phenanthrenes, and fluorenones in its plant tissues [15], which have been indicated to possess antiangiogenic, anticataractogenic, antitumor, antidiabetic retinopathy and antiacetylcholine esterase activities by in vitro and in vivo pharmacological experiments [16]. For example, Erianin, as the main active ingredient isolated from *Dendrobium chrysotoxum*, exhibits potential antioxidant, antiangiogenic, and antitumor activity in various malignancies, including gastric cancer, liver cancer, lung cancer, breast cancer, and osteosarcoma [17]. Additionally, due to the pure bright yellow color, strong fragrance, and long-lasting floral scent in its flowers, *D. chrysotoxum* has ornamental values as great as potted and cut flowers [18]. It is native to Southeast Asian countries and growing naturally in Assam (India), Burma (Myanmar), Bangladesh, Thailand, Vietnam, and Yunnan (China). It prefers cool to warm environmental conditions and is grown at an elevation of 700 to 1100 m [19]. *D. chrysotoxum* produced microscopic seeds fenced within an apparent testa cover. Plants are small sized with clustered clavate and pseudobulbs of 12 to 30 cm in height. The pseudobulbs produce up to 20 flowers with high fragrance but are short lived. The flowers are yellow and have orange-colored lips with pleated edges [20]. In China, in nursery and field conditions, *D. chrysotoxum* plants have been affected by the pathogenic fungi *Pythium vexans*, the causative agent of stem-rot disease [21].

The genus *Fusarium*, a large group of filamentous fungi, belongs to the family Nectriaceae and is pathogenic to plants [22]. Based on its scientific and economic importance, *Fusarium* was recently included in the top-10 most important plant pathogenic fungal genera worldwide [23]. *Fusarium oxysporum* f. sp. *cubense,* the causative agent of banana Panama disease, leads to billions of dollars in losses to farmers, and in case of a severe outbreak, the death of the whole crop occurs [24]. The *Fusarium graminearum* and *Fusarium oxysporum* f. sp. *vasinfectum* cause *Fusarium* head blight (FHB) disease in wheat and *Fusarium* wilt (FW) disease in cotton, respectively, as devastating diseases in the field [25,26]. *Fusarium* as pathogens that cause foliar and root diseases in orchids have been reported, including *Fusarium oxysporum*, *Fusarium proliferatum*, *Fusarium solani*, *Fusarium subglutinans*, and *Fusarium fractiflexum* [27]. The commonly employed methods are the morphological characterization and ITS sequencing identification of the *Fusarium* species complex. *Fusarium* complex is nonspecific and commonly causes root rot, decline in vigor, and wilt symptoms and is responsible for the mortality of taxonomically diverse groups of plants. On the other hand, numerous species of *Fusarium oxysporum* cause seed rot, damping off, and vascular wilt symptoms in vegetables, fruits, and ornamental plants [28]. Wilt-disease complexes have been reported in many vegetables, field crops, plantation crops, fruit trees, medicinal crops, etc., as the pathogen has a wide host range.

In August 2019, leaf-blight symptoms were first observed in the *D. chrysotoxum* nursery at the Dehong Institute of Tropical Agriculture, Ruili City, Yunnan Province, China. This disease causes necrosis and dryness of leaf tips after 7 days of seeding, with a disease incidence of 60%. The disease expansion causes plant leaves to fall off, which limits plant growth and continues to spread over 80% of the plants under high temperatures and humidity, becoming a huge obstacle and seriously threatening the production of *D. chrysotoxum* in Yunnan. This study aimed to isolate and identify the causal agent of leaf blight disease of *D. chrysotoxum* through morphological and molecular techniques, which will help us develop integrated disease management strategies to minimize production losses.

## 2. Materials and Methods

### 2.1. Sample Collection

In August 2019, the samples of *Dendrobium chrysotoxum* (cv. Jin Gu Zi Chui, accession 20140053) showed leaf blight disease symptoms (yellowing of leaves and marginal necrosis). This disease causes necrosis and dryness at the tips of the leaves. Leaves shedding occurs in the later stages of disease development, which can lead to limited plant growth and continues to spread in approximately 80% of plants under high temperature and humidity conditions in the nursery. The diseased leaf samples (yellowing of leaves and marginal necrosis) were collected from the nursery in Ruili City (24° N, 98° E), Yunnan Province, China. Disease samples were kept in the icebox until delivered to the laboratory and stored at 4 °C for isolation of the pathogenic fungi and future use. Healthy *D. chrysotoxum* plants were collected from the Dehong Institute of Tropical Agriculture, Ruili City, to confirm the pathogenicity of the isolates.

### 2.2. Isolation of the Pathogen

The pathogen was isolated from leaf parts, and the diseased leaves were initially washed with sterile water. Then, the margin of diseased leaves was cut into small pieces (0.5 × 2 cm) with a sterilized blade, surface sterilized with a 75% (*v*/*v*) ethanol solution for two minutes and washed thrice with the sterilized distilled water. Three to five pieces from each sample were then placed in the Petri plates containing potato–dextrose–agar (PDA) (potato 200 g/L, dextrose 20 g/L, agar 18 g/L, and pH 7.0) and incubated at 28 °C in the dark. After 5 to 7 days of incubation based on colony morphology, fungal hyphae were picked, placed on PDA plates, purified by a single-spore technique, and stored at 4 °C in PDA slants for future use [29].

### 2.3. Morphological Identification

A single spore was picked with the help of a sterilized needle from the pure culture of each strain, placed in the Petri plates containing PDA as an artificial growth medium, and incubated at 28 °C for 7 to 10 days. Morphological identification was made using different identification keys for *Fusarium* spp., e.g., vegetative growth on culture characteristics, colony color, and reproductive structure. The spore characteristics (shape and size) were observed under an Olympus BX53 microscope (Tokyo, Japan) [28,30].

### 2.4. DNA Extraction

Total genomic DNA was extracted from the 7-to-10-day-old cultures of isolated fungi by scraping the surfaces of growing colonies on PDA. For DNA extraction, fresh mycelium from all four isolates was harvested aseptically. Around 1 g of mycelium was used for DNA extraction using the cetyltrimethylammonium bromide (CTAB) method, as described by Brandfass’s report [31]. The concentration and quality of extracted DNA was checked using a NanoDrop spectrophotometer (ND2000, Thermo Scientific, Wilmington, WA, USA) and by gel electrophoresis in 1.0% (*w*/*v*) agarose gel prepared in 1 × TAE buffer solutions, and the electrophoresis was run for 25 min at 160 V. The DNA was then stained with ethidium bromide (2 mg/L), and the gel was visualized under ultraviolet (UV) light (Bio-Rad Gel Doc™ XR Imaging system, Wilmington, WA, USA) [32]. The extracted DNA was stored at −20 °C for future use.

### 2.5. PCR Amplification, Molecular Identification, and Construction of Phylogenetic Tree

The isolated strains were identified by PCR amplification of four different genes, including the internal transcribed spacer (*ITS*) specific region of rDNA, the largest subunit of RNA polymerase Ⅰ (*RPB1*), the second-largest subunit of RNA polymerase Ⅱ (*RPB2*), and translation elongation factor (*EF-1α*) were amplified using four corresponding primer pairs ITS1/ITS4, EF1/EF2, F7/R8, 5F2/7CR. The primers used in this study to amplify ITS, *RPB1*, *RPB2*, and *EF-1α* genes are listed in Table 1 [33,34,35]. All PCRs were performed in a 25 μL reaction mixture containing 12 μL 2×EasyTaq^®^ PCR SuperMix (Takara Biotechnology Co., Ltd., Dalian, China), 100 ng of genomic DNA as template, 1 μL of each forward and reverse primers (10 uM), and ddH_2_O adjusted to the final volume. The PCR amplification conditions were as follows: initial denaturation at 94 °C for 5 min, followed by 35 amplification cycles of denaturation at 94 °C for 30 s, annealing at 55 °C for 30 s, extension at 72 °C for 1 min, and final extension at 72 °C for 7 min.

The PCR amplicon products were visualized by the gel electrophoresis in 1.0% (*w*/*v*) agarose gel and sent to the company (TSINGKE^®^ Co. Ltd., Beijing, China) for sequencing by the Sanger method. The sequences generated in this study were subjected to a BLAST search in the GenBank nucleotide database (http://www.ncbi.nlm.nih.gov/BLAST, accessed on 14 February 2021), and sequences were deposited in the NCBI GenBank. The phylogenetic trees based on sequences of 4 genes (*ITS*, *RPB1*, *RPB2*, and *EF-1α*) were constructed separately using the aligned nucleotide sequences with 1000 bootstrap replicates (removing gaps) following the maximum likelihood tree phylogeny of MEGA software version 7.0.21. Further, the DNA sequences based on the MYCOBANK online Fusarium MLTS database (https://fusarium.mycobank.org/, accessed on 22 March 2022) were accessed for species identification [36].

### 2.6. Pathogenicity Test

According to Koch’s postulates, a pathogenicity test was performed to confirm the pathogenicity of 4 isolated strains. Spore suspension of all four isolated strains was prepared according to Han’s protocols [37]. The 4 isolates suspension was obtained from 7-to-10-day-old cultures grown on a PDA medium, prepared from the actively grown mycelium of the 7-day-old culture in 15 mL sterilized distilled water in each of the Petri dishes, and a sterile inoculation needle was used to scrape the mycelium on the medium surface. Mycelium was harvested aseptically from the medium’s surface, and spore suspension was adjusted to 1 × 10^6^ conidia/mL using a hemocytometer. The collected spores were observed under an optical microscope (Olympus BX53 microscope, Tokyo, Japan). Two-month-old *D. chrysotoxum* (cv. Jin Gu Zi Chui) plants were sprayed with a 20 mL, 1 × 10^6^ conidia/mL spore suspension, whereas the control plants were treated with sterilized ddH_2_O (water control). The treated plants were kept in the greenhouse at 28 ± 2 °C and 75% relative humidity [38]. The experiment was repeated thrice for each isolated strain, with five plants in each treatment as replicates.

### 2.7. Pathogen Reisolation

For pathogen reisolation, the leaf samples from the water control plants and the pathogen-inoculated plants were prepared, incubated, and mycelia subcultured on PDA as to the previous process. DNA was extracted from the reisolated pathogens, and PCR products were sent for Sanger sequencing at TSINGKE^®^ Co. Ltd. (Kunming, China). A BLAST search of the resulting *ITS* and *EF-1α* sequences was performed using the NCBI database.

## 3. Results

### 3.1. Nursery Observations and Isolations

**Figure 1:**
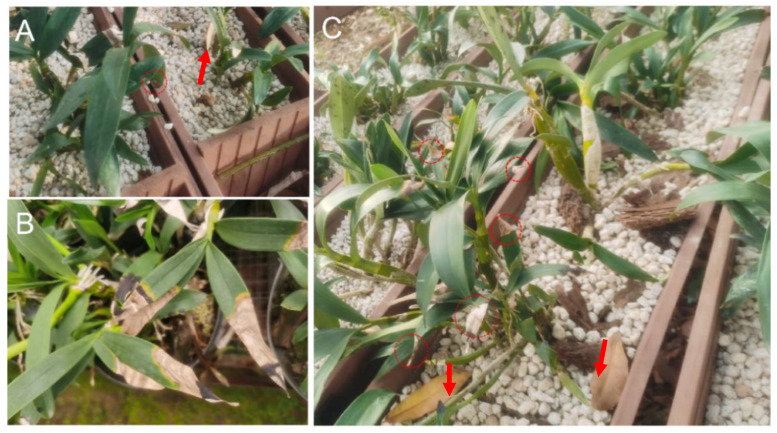
Symptoms of leaf blight on *D. chrysotoxum* seedlings in the nursery beds. (**A**) Early water-soaked corrugated lesions on the stem and graft interface; (**B**) yellowing of leaves and marginal necrosis; (**C**) plant leaves fall off. Arrows indicated leaves fall off the stem; Circles indicated lesions in (**A**,**C**).

In August 2019, a disease survey was conducted on *D. chrysotoxum* (cv. Jin Gu Zi Chui) nurseries (110 seedbeds) in four greenhouses at the Dehong Institute of Tropical Agriculture, Ruili City. After 7 days of seeding, as the disease initiated, necrotic water-soaked corrugated lesions were first observed on the tips of blades (Figure 1A), and later, the necrotic lesion expanded to over half of the leaf blade with a yellow border (Figure 1B). In case of a severe attack, the disease expansion causes plant leaves to fall off (Figure 1C). During the wet summer, the disease incidence rapidly increased, which affected more than 60% of the plants in the nursery. Four isolates, DHRL-01, DHRL-02, DHRL-03, and DHRL-04, were isolated from the infected leaves of *D. chrysotoxum.* Morphological and molecular characterization were performed for the identification of the isolated pathogen.

### 3.2. Morphological Identification

**Figure 2:**
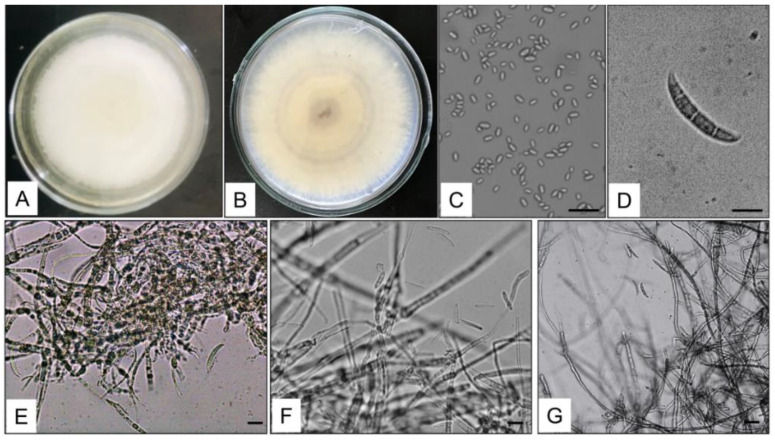
Morphological characteristics of *Fusarium oxysporum* isolated from diseased *Dendrobium chrysotoxum* plants. The colony growth on PDA medium (**A**, **B**), microconidia (**C**), macroconidia (**D**), and conidia chain bar = 10 μm and macroconidia (**E**–**G**).

The four isolated strains were grown on a PDA medium for seven days for morphological identification. The strain colonies were characterized by an abundant white cottony mycelium and a dark-purple undersurface on the PDA medium (Figure 2A,B). Strains produced three types of spores: microconidia, macroconidia, and chlamydospores. Microconidia were oval to ellipsoid or renal shaped, without septation, and were 2.57–3.38 × 5.25–10.62 µm in size (Figure 2C). Macroconidia were sickle shaped, tapering at both ends, having 2–4 septation, and 19.32–36.55 × 3.44–5.52 µm in size (Figure 2D), while the chlamydospores were produced in the chain (Figure 2E–G). The four isolated strains were identified as *Fusarium* spp. based on colony morphology.

### 3.3. Molecular Identification

**Figure 3:**
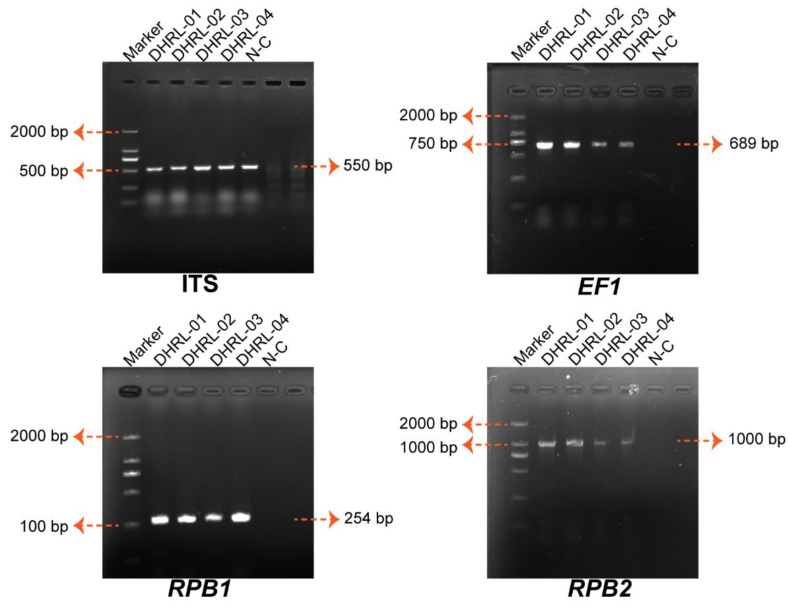
One percent agarose gel image of PCR-amplified products of *ITS*, *EF-1α*, *RPB1*, and *RPB2* gene of four isolates from *Dendrobium chrysotoxum*. Marker—2000 bp DNA marker (D2000, Tiangen, Beijing, China), DHRL-01~DHRL-04 were the gene PCR production of 4 isolates, and N-C—negative control.

Molecular identification of isolated strains was done by the PCR amplification of ITS, *EF-1α*, *RPB1*, and *RPB2* genes. Through PCR amplification, the results in a product were obtained at 527–550 bp (*ITS*), 687–689 bp (*EF-1α*), 254–256 bp (*RPB1*), and 995–1018 bp (*RPB2*) for all isolated strains (Figure 3). The isolated strains were identified as having 99.42–99.62% similarity with the sequences of *Fusarium oxysporum* in the NCBI database, and all sequences are deposited in the NCBI GenBank (Table 2).

**Figure 4:**
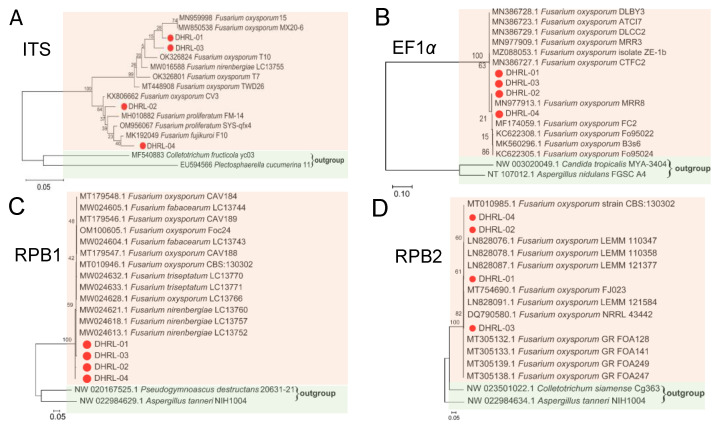
Maximum-likelihood phylogenetic tree generated from *ITS* (**A**), *EF-1α* (**B**), *RPB1* (**C**), and *RPB2* (**D**) sequences analysis of isolated strains of DHRL-01~04, respectively. *Colletotrichum fructicola* and *Plectosphaerella cucumerina* were used as the outgroup of ITS. *Colletotrichum fructicola* and *Aspergilus nidulans* were used as the outgroup of *EF-1α*, *Pseudogymnoascus destructans,* and *Aspergilus tanneri* were used as the outgroup of *RPB1, Aspergilus tanneri* and *Aspergilus tanneri* were used as the outgroup of *RPB2*. Scale bars indicate the number of substitutions per site. Bootstrap values are expressed as percentages based on 1000 replicates. Different background colors represent different clusters in the phylogenetic tree.

The phylogenetic tree was constructed for each gene, *ITS*, *EF-1α*, *RPB1,* and *RPB2*, to confirm the NCBI blast results (Figure 4). The phylogenetic tree results based on *ITS* showed that four isolated strains were grouped in one clade with reference sequences of *Fusarium* spp. strains, supported by a 100% bootstrap value, but bootstrap values equal to or lower than 64% are shown as isolated strains clustered together as one with *Fusarium oxysporum* (Figure 4A). The results of phylogenetic trees based on three genes, *EF-1α*, *RPB1*, and *RPB2* sequences, showed all four isolates (DHRL-01~04) were clustered within the clade corresponding to the species *Fusarium oxysporum* with 100% bootstrap support (Figure 4B–D). Further research showed the species of the four isolates to be confirmed by the MLST database; the MLST was determined based on polyphasic identification using the *Fusarium* MLST database, and the identification result shows four isolates have 100% MLST similarity to the *Fusarium oxysporum* species complex. In total, we confirm the four isolates were *Fusarium oxysporum* isolated from *Dendrobium chrysotoxum* plants.

### 3.4. Pathogenicity Test

**Figure 5:**
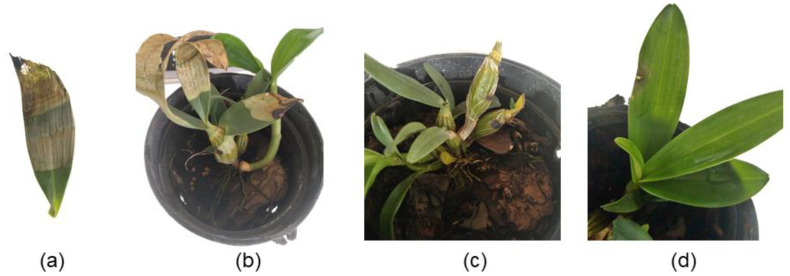
Symptoms produced on *Dendrobium chrysotoxum* plants inoculated with *Fusarium oxysporum.* (**a**,**b**) yellowing, marginal necrosis, and, later, expansion to the bottom leaves after 15 days post-inoculation with *Fusarium oxysporum* DHRL-01. (**c**) Whole leaves became blighted and dropped after 30 days post-inoculation with *Fusarium oxysporum* DHRL-01, and (**d**) healthy plants as control (inoculated with sterilized ddH_2_O) was only physical damage in leaves.

A pathogenicity test was performed to fulfill Koch’s postulates and confirm the virulence of the four isolated strains. Figure 5 shows that the symptoms developed within 30 days after inoculation of the isolate DHRL-01. The disease symptom appeared first on the top leaves of plants as vein clearing, yellowing, and marginal necrosis and later expanded to the bottom until the blight symptoms showed on the whole blade (Figure 5a–c), while the control plants remained healthy (Figure 5d). The other three isolates also caused similar symptoms in *D*. *chrysotoxum* leaves that were inoculated with the spore suspension. The pathogenicity test results show that four isolates were observed to be pathogenic by producing symptoms identical to those observed on the naturally infected plants.

### 3.5. Pathogen Reisolation

After 15 days post-inoculation, we reisolated the four pathogens from leaf samples of pathogen-inoculated plants. The results showed that the same morphological feature in the growth media (PDA: potato–dextrose–agar media) were reisolated from the leaves and were inoculated with pathogens, and a BLAST search of ITS and EF-1α sequencing results performed using the NCBI database showed that reisolated pathogens were the same as the inoculated pathogens with 100% identity, completing Koch’s postulates.

## 4. Discussion

Some *Dendrobium* plants are considerably threatened by destruction due to many disease-causing microorganisms [39]. *Dendrobium chrysotoxum*, an economically important medicinal plant, is mainly planted in Yunnan province. However, the leaf-blight disease reduces its production and may be destructive to the medical and esthetic values of the *D. chrysotoxum plant*. We applied a polyphasic approach to identify fungal isolates associated with the blight disease of *D. chrysotoxum* at the seeding stage in a nursery room in Ruili City, China. A combination of morphological characteristics and molecular phylogeny results identified the fungal isolates as *Fusarium oxysporium* (Figure 2 and Figure 4).

The genus *Fusarium* comprises the most important and notorious fungi, consisting of diverse species of complex and economically destructive phytopathogens associated with leaf-blight diseases on a wide range of host plants, for example, banana leaf-blight disease, peanut leaf blight, and plum leaf blight [40,41,42]. Some *Dendrobium* plants have been reported as disease hosts caused by *Fusarium* spp., for example, dieback disease [43] and soft rot disease of *D. officinale* in China [44] and leaf blotch of *D. antennatum* in Malaysia [45]. Still, there are no records of its occurrence in *D. chrysotoxum* plants. Therefore, this is the first report of *Fusarium* spp., causing leaf-blight disease in *D. chrysotoxum* seedlings in China. In this study, four strains of *Fusarium oxysporium* DHRL-01~04 were isolated from the diseased leaves of *D. chrysotoxum*.

Additionally, identification and pathogenicity assays were performed for the *F. oxysporum* associated with the leaf-blight disease of *D. chrysotoxum*. The pure culture characteristic of *Fusarium oxysporum* produced white-to-pale cottony mycelium, and a dark-purple undersurface on the PDA medium was accordingly from *Fusarium* spp. The morphotype of the fungus was examined with microscopy. The sickle shaped, tapering at both ends having septation, was oval to ellipsoid or renal-shaped microconidia and macroconidia, and the shapes and sizes of the microconidia and macroconidia were observed as *Fusarium* spp. reported in the earlier studies [46,47].

The *Fusarium* species were isolated based on morphological features alone and confronted several discrepancies, as frequently observed in previous studies [48]. Currently, the species identified by the PCR assay and sequence are required for accurate taxonomical characterization, including the *Fusarium* species [35]. Sequences of the ITS region have been commonly employed in the phylogenetic analysis of fungus genera at the species level [49]. Based on *ITS* sequences, the isolated strains were identified as *Fusarium oxysporum* species (Figure 3A). However, relying only on a single gene for phylogenetic analysis is often uninformative at the species level and aligned across the members of complex or closely related species [50]. The *EF1-α* gene encodes an essential part of the protein translation machinery, and non-orthologous copies of the gene have not been discovered in the genus to be as helpful and informative for identifying *Fusarium* until the species level. Many previous studies have reported the *EF1-α* gene was used to identify *Fusarium* species [51,52]. The *RPB1* and *RPB2* genes have highly variable introns that can only be aligned reliably across members of a species complex or several closely related ones; the portions of *RPB1* and *RPB2* sequenced can easily be aligned across *Fusarium* [35]. Therefore, in addition to the morphological features and *ITS* sequencing, the wilt disease associated with *Fusarium* sp. was characterized based on partial *EF-1α*, *RPB1*, and *RPB2* sequencing. The sequence-based phylogenetic trees provide essential information on the systematics of the *Fusarium* species complex. So, we further constructed a phylogenetic tree for *EF-1α*, *RPB1,* and *RPB2* gene sequences for four strains DHRL-01~04. Further on in the phylogenetic analysis, the sequence of *ITS*, *EF-1α*, *RPB1,* and *RPB2* genes used in the *Fusarium* MLTS database (http://www.westerdijkinstitute.nl/fusarium/, accessed on 22 March 2022) also demonstrated that the sequenced four strains DHRL-01~04 in our study were identified as *Fusarium oxysporum* species (similar by more than 99%), causing the leaf-blight disease of *D. chrysotoxum*.

Leaf-blight disease management required multifaceted approaches, and *D. chrysotoxum* as a medicinal plant needed more attention. It is widely grown in the western part of the country, and very limited fungicides are advised to manage diseases. The excessive use of fungicides harms human health and is environmentally unfriendly [53]. During the crop cycle, leaf-blight disease occurs at any stage of the crop, from seedling to plant maturity. But, younger plants at the nursery, especially in the seeding stage, are more affected by this disease, and seedlings cannot be transplanted. So, considering the above facts, further studies should be needed to determine the appropriate environmental conditions for disease development and how disease spreads.

Chemical soil fumigation and resistant cultivars were employed as the main measures to control the *Fusarium* wilt disease. However, the broad-spectrum chemical fungicides used to fumigate soil before planting are environmentally damaging [54]. Disease-resistant planting is the most cost-effective, environmentally safe method of controlling wilt disease; breeding for resistance can be difficult when no dominant gene is known [55]. Biological control has been considered an alternative strategy to chemical germicides to control the *Fusarium* wilts by using antagonistic nonpathogenic microorganisms that have the potency to minimize or recover the harmful effects in numerous crops. Recently, plant-growth-promoting fungi (PGPF), rhizobacterium (PGPR), and arbuscular mycorrhizal fungi (AMF) as biocontrol agent managers of *Fusarium* wilt disease for crop plants have been heavily reported [56,57]. Additionally, disease-suppressive specific endophytes are used as potential biocontrol agents to manage the disease [58]. In recent years, research on *Fusarium* taxonomy in China has developed rapidly based on morphology and molecular biology. This experiment targets field prevention and treatment of *D. chrysotoxum* leaf-blight disease. It provides a reference for further genetic analysis and cultivation of disease-resistant varieties of *D. chrysotoxum*.

## 5. Conclusions

In conclusion, based on morphological characterization and molecular phylogenetic analysis by genes ITS, *EF-1α*, *RPB1*, and *RPB2*, the isolated strains were identified as *Fusarium oxysporum*. We concluded that *F. oxysporum* causes leaf-blight disease in *D. chrysotoxum* in China. This is the first study of pathogenic fungi causing leaf-blight disease in Chinese *D. chrysotoxum*. However, further study should be done to identify the specific disease-suppressive biocontrol agent to control the incidence of *F. oxysporum* leaf-blight disease.

## Figures and Tables

**Table 1:** Primers used in this study.

Gene	Primers	Sequence (5′–3′)	Annealing Temperature (°C)
*ITS*	ITS1-F	TCCGTAGGTGAACCTGCGG	55
	ITS4-R	TCCTCCGCTTATTGATATGC	
*EF-1α*	EF1-F	ATGGGTAAGGARGACAAGAC	55
	EF2-R	GGARGTACCAGTSATCATG	
*RPB1*	F7-F	CRACACAGAAGAGTTTGAAGG	55
	R8-R	CAATGAGACCTTCTCGACCAGC	
*RPB2*	5F2-F	GGGGWGAYCAGAAGAAGGC	55
	7CR-R	CCCATRGCTTGYTTRCCCAT	

**Table 2:** Nucleotide BLAST results from the *Fusarium* MLST databases for the FOSC GenBank and accession numbers of the *Fusarium oxysporum* isolated from *Dendrobium chrysotoxum* plants.

Isolate	Fusarium MLST (Number)	Fusarium MLST Similarity %	GenBank Accession Number			
			ITS	EF-1α	RPB1	RPB2
DHRL-01	*Fusarium oxysporum* species complex (NRRL 22549)	100	MW599746	MW703468	MW703472	MW703476
DHRL-02	*Fusarium oxysporum* species complex (NRRL 22549)	100	MW599747	MW703469	MW703473	MW703477
DHRL-03	*Fusarium oxysporum* species complex (NRRL 22549)	100	MW599748	MW703470	MW703474	MW703478
DHRL-04	*Fusarium oxysporum* species complex (NRRL 22549)	100	MW599749	MW703471	MW703475	MW703479

## Data Availability

Data are contained within the article.
